# Evaluation of physicochemical properties and antioxidant activities of kombucha “Tea Fungus” during extended periods of fermentation

**DOI:** 10.1002/fsn3.605

**Published:** 2018-02-20

**Authors:** Hashani Amarasinghe, Nimsha S. Weerakkody, Viduranga Y. Waisundara

**Affiliations:** ^1^ Department of Agricultural Plantation Engineering Faculty of Engineering Technology Open University of Sri Lanka Nugegoda Sri Lanka; ^2^ Department of Food Technology Faculty of Technology Rajarata University of Sri Lanka Mihintale Sri Lanka

**Keywords:** antioxidant activity, fermentation, Kombucha, orac, tea, total phenolics content

## Abstract

Kombucha fermentation is traditionally carried out by inoculating a previously grown tea fungal mat into a freshly prepared tea broth and incubating under aerobic conditions for 7–10 days. In this study, four kombucha beverages were prepared by placing the tea fungal mats in sugared Sri Lankan black tea at varying concentrations for a period of 8 weeks. The antioxidant activities, physicochemical, and qualitative properties were monitored prior to the commencement of the fermentation process, one day after the inoculation with the microorganisms and subsequently on a weekly basis. All samples displayed a statistically significant decrease (*p *<* *.05) in the antioxidant activity at the end of 8 weeks, which was indicative of the decreasing functional properties of the beverage. The physicochemical properties indicated increased acidity and turbidity, which might decrease consumer appeal of the fermented beverage. Further studies are necessary to test the accumulation of organic acids, nucleic acids, and toxicity of kombucha on human organs following the extended period of fermentation.

## 1 INTRODUCTION

Kombucha is a fermented beverage obtained through the fermentation of sugared tea (*Camellia sinesis*) with symbiotic bacteria and osmophilic yeast. The word “Kombucha” is derived from the Japanese words “seaweed” (Kombu) and “tea” (cha) (Ernst, 2003). Kombucha tea is also known as red tea fungus, Champignon de longue vie, Chainii grib, Ling zhi, kocha kinoko, and Chainii kvass (Hara, Luo, Wickremashinghe, & Yamanishi, 1995; Malbaˇsa, Lonˇcar, Vitas, & ˇCanadanovi′c‐Brunet, 2011; Watawana, Jayawardena, Gunawardhana, & Waisundara, 2015). It is believed that kombucha originated in China over 2,000 years ago (Martin et al. 1995; Liu et al. 1996; Srihari & Satyanarayana, 2012), while many other historical reports indicate that this beverage was consumed in countries such as Russia, Germany, and the Middle East as well (Dipti, Yogesh, & Kain, 2003). The kombucha fermentation is traditionally carried out for 7–10 days in household preparation conditions. As the fermentation progresses, the taste of kombucha tea changes from a pleasurably fruity, sour, and sparkling flavor to a mild vinegar‐like taste, thus increasing the consumer acceptability of the flavor and other sensory aspects of the beverage (Goh et al., 2012; Marsh, Sullivan, Hill, Ross, & Cotter, 2014; Watawana et al., 2015). Ultimately, a pleasantly sour, slightly sparkling, apple cider‐like beverage is produced. It can be produced at home by fermentation using mail order fungus or a tea fungal mat which is domestically prepared. Although green tea can be used for the preparation of this beverage, the combination of black tea and white sugar is considered the finest substrates. The beverage is well‐known to possess many prophylactic and therapeutic benefits, where it is believed to help with digestion, give relief against arthritis, act as a laxative, prevent microbial infections, combat stress and cancer, provide relief against hemorrhoids, impart a positive influence on the cholesterol levels, and facilitate excretion of toxins as well as blood cleansing (Dufresne & Farnworth, 2000; Jayabalan, Radomir, Malbasa, Loncar, & Vitas, 2014; Malbaˇsa et al., 2011). It is also known to help balance the gastrointestinal microbial flora in humans, by acting as a probiotic drink (Kabiri, Setorki, & Darabi, 2013; Malbaˇsa et al., 2011). Furthermore, it is believed to have the ability to improve the health of hair, skin, and nails, reduce stress and nervous disturbances, reduce insomnia, relieve headaches, reduce the craving for alcohol of an alcoholic person, prevent the formation of bladder infections, reduce the kidney calcification, decrease menstrual disorders and menopausal hot flashes, improve eyesight, cellular regeneration, and stimulation of glandular systems in the body, relieve bronchitis and asthma, and enhance the general metabolism (Jayabalan et al., 2014; Mayser et al., 1995; Morshedi & Dashti‐Rahmatabadi, 2010).

During the kombucha fermentation, many compounds with radical scavenging properties such as polyphenols are released from the tea (Malbaˇsa et al., 2011). Polyphenols have the ability to scavenge free radicals, in particular reactive oxygen species (ROS), which are considered to possess high levels of wide‐ranging antioxidant properties (Srihari & Satyanarayana, 2012). The presence of tea polyphenols is known to impart the antioxidant activity to the kombucha as well (Jayabalan et al., 2014). Studies examining kombucha tea prepared using substrates such as green tea, black tea, and tea waste material have been shown to have a high radical scavenging activity (Jayabalan, Subathradevi, Marimuthu, Sathishkumar, & Swaminathan, 2008). However, the total antioxidant activity depends on the fermentation time, type of the tea material, and the microbiota present in the tea fungal mat (Jayabalan et al., 2014; Sievers et al., 1995; Sreeramulu et al., 2000). Although the tea fungus has the ability to enhance the radical scavenging activity by the fermentation process, whether these beneficial effects of the beverage are enhanced through extended periods of fermentation remains a question. To address this issue, this study focused on monitoring the changes to the antioxidant activities and physicochemical effects of kombucha “Tea Fungus” during an extended fermentation period of 8 weeks. The fermentation process has not been monitored for such a lengthy duration, and as such, the antioxidant properties of the beverage have not been evaluated for such a period as well. It is of interest to see whether the functional properties of the beverage as well as other physicochemical aspects are altered beyond the typical 7–10 days of fermentation. Most of the physicochemical properties evaluated in this study are related to the mouthfeel and appearance of the beverage. These sensory aspects are important above all as being a fermented beverage, consumers may tend to judge the drink mostly based on the color, clarity, and flow properties.

## 2 MATERIALS AND METHODS

**Figure 1:**
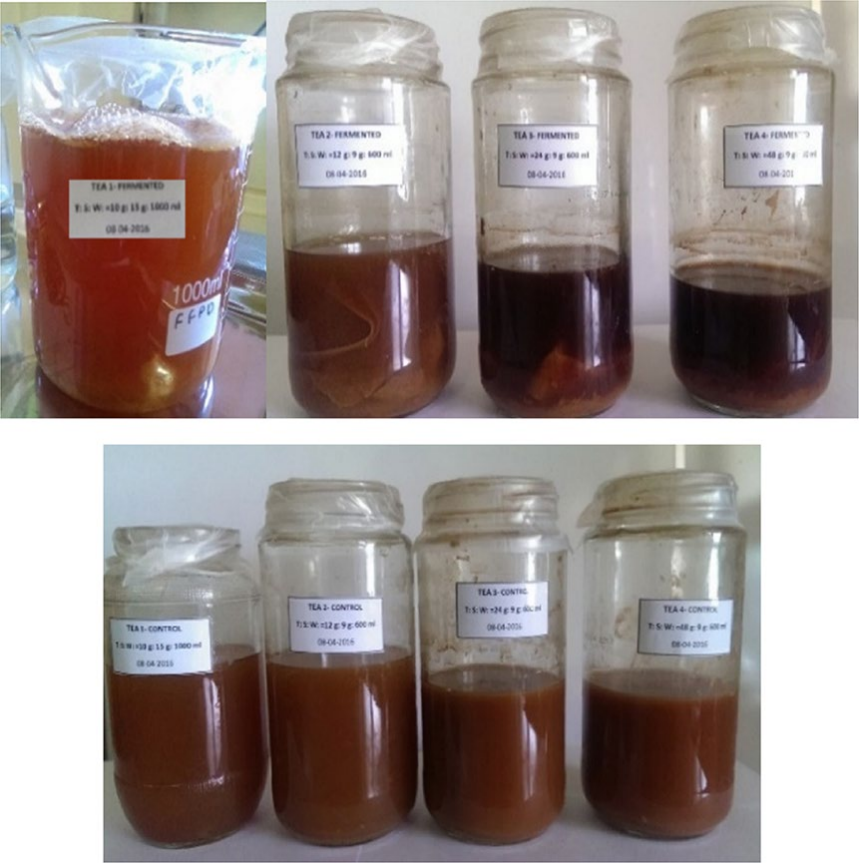
Fermented samples (from top left to right): Tea 1—F, Tea 2—F, Tea 3—F, Tea 4—F; control samples (from bottom left to right): Tea 1—C, Tea 2—C, Tea 3—C, Tea 4—C

The kombucha tea fungal mat was originally obtained from the Yunnan Province of China, following which it was domestically grown in Singapore until three “daughter” mats were developed. The third “daughter” mat was used for this experiment. The dominant acetic acid bacterial species found in the tea fungal mat were *Acetobacter xylinum*,* A. xylinoides*,* A. aceti*,* A. pausterianus,* and *Bacterium gluconicum*. *Kloeckera spp., Schizosaccharomyces pombe, Saccharomyces ludwigii, S*. *cerevisiae, Torulaspora spp., Zygosaccharomyces bailii, and Pichia spp.,* were the dominant fungal species. Sugared black tea was prepared by addition of Sri Lankan black tea into boiling water and allowed to infuse for 5 mins. The black tea used for this experiment was obtained from Kenilworth Estate in Ginigathena, which is one of the tea estates of Watawala Plantations in Sri Lanka. Different tea concentrations were prepared by changing the amount of Sri Lankan black tea. The concentrations of tea in the experimental samples which were prepared are shown in Table 1. The infusion was filtered using a sterile sieve. To this, brown sugar as amounts displayed in Table 1 was added and dissolved. The extracts were left to cool to room temperature. The tea was poured into a sterile glass jar, and the tea fungal mats were added to the fermenting tea samples. Throughout the duration of fermentation, beverages were covered with a Parafilm to avoid contamination from external microorganisms and other types of environmental debris. Representative images of the prepared samples are shown in Figure 1. The physicochemical and antioxidant parameters were evaluated according to the methodologies mentioned in the subsequent sections, during the time points prior to inoculation of the tea with the tea fungus, 1 day after the fermentation, followed by weekly analysis for a total period of 8 weeks. With the exception of the weight of the tea fungal mat, all other parameters were measured in the broth of the fermented samples.

**Table 1:** Ingredients used for the preparation of the various concentrations of tea samples

Tea Sample	Black Tea (g)	Brown sugar (g)	Water (ml)
Tea 1—F (Fermented)	10	15	1,000
Tea 1—C (Control—Unfermented)	10	9	600
Tea 2—F (Fermented)	12	9	600
Tea 2—C (Control—Unfermented)	12	9	600
Tea 3—F (Fermented)	24	9	600
Tea 3—C (Control—Unfermented)	24	9	600
Tea 4—F (Fermented)	48	9	600
Tea 4—C (Control—Unfermented)	48	9	600

### 2.1 pH value and total soluble solids (TSS) of Kombucha

The pH values were measured using an electronic handheld pH meter (Testo 206 PH1, Keison, London, UK). The TSS were measured with a hand refractometer (ERMA Inc. A—Contrast II—520 –0, Tokyo, Japan).

### 2.2 Change in weight of kombucha mat and clarity

Observations for mat formation and changes in clarity (clear/turbid) were recorded on a weekly basis. In addition, the percentage of weight gains or loss of the tea fungus mat (mother culture + daughter cultures) between day 0 (before the initiation of the fermentation process) and the day of data collection were calculated based on the differences in the wet weights of the total tea fungal mat in the broth.

### 2.3 Total phenolics content (TPC)

The method as described by Huang, Ou, Hampsch‐Woodill, Flanagan, and Deemer (2002) was used for determining the TPC values using the Synergy™ HTX Multi‐Mode Microplate Reader (BioTek Instruments, Winooski, VT, USA) with Gen5™ software. Results were expressed as micrograms gallic acid equivalents per ml (μg GAE ml^−1^).

### 2.4 Oxygen radical absorbance capacity (ORAC) assay

The ORAC assay was carried out according to the method by Prior et al. (2003) using the Synergy™ HTX Multi‐Mode Microplate Reader (BioTek Instruments, Winooski, VT, USA) with Gen5™ software. The antioxidant capacity of the tea samples was measured in terms of micromole Trolox equivalents per ml (μM TE ml^−1^). Fluorescein disodium was used for the kinetic monitoring of free radical quenching, and 2, 2‐azobis (2‐amidinopropane) dihydrochloride (AAPH) was used as the free radical source. The excitation and emission wavelengths were 485 nm and 528–538 nm, respectively.

### 2.5 DPPH radical scavenging assay

The DPPH radical scavenging activity assay was carried out using the Synergy™ HTX Multi‐Mode Microplate Reader (BioTek Instruments, Winooski, VT, USA) with Gen5™ software. Extract concentrations of 62.5, 125, 250, 500, and 1,000 ppm were prepared by dilution with 75 mM phosphate buffer (pH = 7.4). The microplate was incubated at 37°C for 30 min, and the absorbance was measured at 517 nm. Each sample concentration was added in triplicate into the microplate. The antioxidant activity was calculated as % DPPH radical scavenging activity, by substituting the absorbance values into the following equation:

$$\%\text{DPPH}\;\text{radical}\;\text{scavenging activity}=\frac{({\text{Abs}}_{\mathrm{Control}/\mathrm{Extract}}-{\text{Abs}}_{\mathrm{Blank}})\times 100}{{\text{Abs}}_{\mathrm{Control}}}$$

### 2.6 *Statistical analysis*

All data were presented as means (± standard deviation) of at least three independent experiments (*n* ≥ 3), with each experiment having a minimum of three replicates of each sample. For comparisons between samples, data were analyzed by ANOVA using the software Minitab version 17. A probability of 5% or less was considered as statistically significant.

## 3 RESULTS AND DISCUSSION

### 3.1 *pH and total soluble solids*

A statistically significant decrease (*p *<* *.05) was observed in the overall pH of all kombucha samples with the fermentation time as compared to Tea 1—F. There were no statistically significant differences (*p *<* *.05) of pH between control and fermented samples on day 0, week 2, and week 3, but a statistically significant difference in pH (*p *<* *.05) between kombucha samples was observed in week 5. The initial pH of the tea samples was 5.3, and it dropped to 4.3 during the fermentation period. It showed a rapid decrease until 2 weeks of fermentation and continued to decrease to a certain extent up to 8 weeks. Studies show that the pH of kombucha at drinkable level decreases from around 5.0 to 2.5 within 6 days of fermentation (Liu et al. 1996; Greenwalt, Ledford, & Steinkraus, 1998). Similarly, in this study, the pH decreased from 5.6 to 3.6 within the first week (7 days) of fermentation in the control sample. According to the study of Chen and Liu (2000), the final pH value of the liquid broth after 30 days was 2.5, which was much lower than the pH for optimum growth (pH 5.4–6.3) of yeasts. The decrease in pH value could be due to the increased concentration of organic acids produced during the fermentation process by bacteria and yeasts in the tea fungal mat, which then seeped into the broth.

It was also observed that the pH and TSS of the broths of the control samples also changed throughout the period of analysis. While it may be interpreted that these changes would make the interpretation of fluctuations in the experimental samples difficult, the purpose of the control is to observe the physiological behavior of tea samples to which the kombucha fungal mats have not been added. Additionally, the changes in the pH and TSS in the control samples may not have necessarily been due to contamination, as given the total period of analysis of 2 weeks, it is possible that the beverage itself underwent changes in viscosity due to exposure to oxygen, and therefore as a result of oxidation. Attempts were made, nevertheless, to minimize any such exposure or contamination, despite bearing in mind the inevitable.

### 3.2 Weight of the kombucha mat and clarity

**Figure 2:**
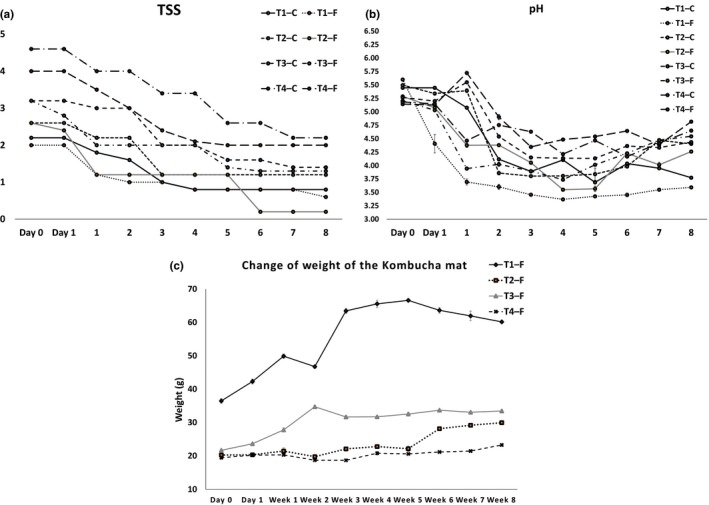
Changes to the (a) TSS, (b) pH contents, and (c) weight of the kombucha mat with fermentation time. Error bars represent the SEM

Changes to the weight of the kombucha mat are presented in Figure 2c. The final weights of the kombucha mats were statistically significantly different (*p *<* *.05) as compared to their initial weights. The weight of the kombucha mat of Tea 1—F increased with time to some extent and continued to statistically significantly decrease (*p *<* *.05) at a lower rate after week 5 compared with the other kombucha samples. The weight of Tea 2—F and Tea 3—F statistically significantly increased (*p *<* *.05) with fermentation. Although an increase in weight was observed in Tea 4—F, it was not statistically significant (*p *<* *.05). The mean separation weight of the kombucha mats of Tea 2—F and Tea 3—F in weeks 1, 2, 4, and 5 was not statistically significantly different (*p *<* *.05). The weight of the kombucha mat increased in all samples except Tea 1—F. This may be due to the growth of bacteria and yeast of the tea fungus and reduction in required TSS for the growth of fungus with the prolonged time of 8 weeks. Observations of clarity with time are presented in Table 2. The clarity of kombucha beverage changed from clear to turbid during the 8 weeks of fermentation period. Both clarity and the changes to the weight of the mat are related to the amounts of cellulose produced by the acetic acid bacteria present in the tea fungal mats. Increased turbidity would indicate a higher amount of cellulose and other fibrous matter being released to the broth.

**Table 2:** Changes to the clarity of kombucha samples and controls

	T1—F	T1—C	T2—F	T2—C	T3—F	T3—C	T4—F	T4—C
Day 0	Very clear	Very clear	Very clear	Very clear	Very clear	Very clear	Very clear	Very clear
Day 1	Clear	Very clear	Clear	Very clear	Very clear	Very clear	Very clear	Very clear
Week 1	Turbid	Very clear	Turbid	Very clear	Turbid	Clear	Turbid	Clear
Week 2	Turbid	Clear	Turbid	Clear	Turbid	Clear	Turbid	Clear
Week 3	Turbid	Clear	Turbid	Clear	Turbid	Turbid	Turbid	Turbid
Week 4	Turbid	Turbid	Turbid	Turbid	Very turbid	Turbid	Very turbid	Turbid
Week 5	Turbid	Turbid	Turbid	Turbid	Very turbid	Turbid	Very turbid	Turbid
Week 6	Very turbid	Turbid	Very turbid	Turbid	Very turbid	Very turbid	Very turbid	Very turbid
Week 7	Very turbid	Turbid	Very turbid	Turbid	Very turbid	Very turbid	Very turbid	Very turbid
								
Week 8	Very turbid	Turbid	Very turbid	Turbid	Very turbid	Very turbid	Very turbid	Very turbid

Similar to the parameters of pH and TSS, it was observed that the clarity of the control samples also changed during the period of analysis from “very clear” to “turbid.” As in the instance of the previous parameters, although it may be interpreted as a evidence of contamination, this may not have necessarily been the case for the same reason as oxidation and other biochemical changes take place within the tea itself, through exposure to air—although attempts were made as much as possible to minimize such activity.

### 3.3 *TPC assay, ORAC assay, and DPPH radical scavenging assay*

**Figure 3:**
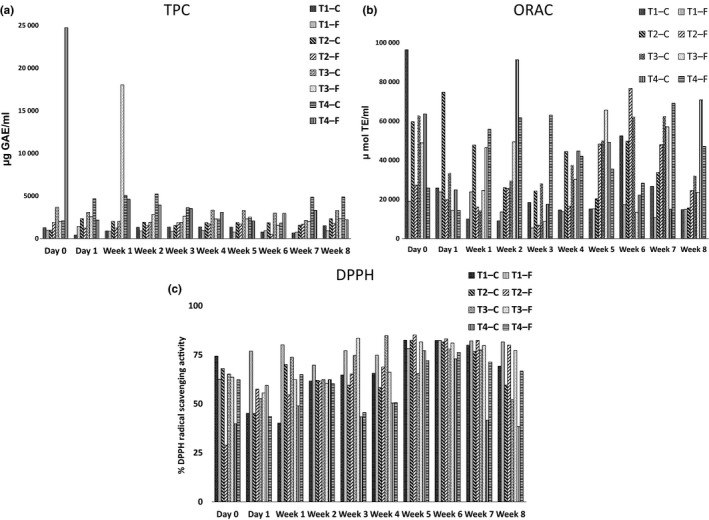
(a) Total phenolic content, (b) ORAC, and (c) DPPH scavenging activities of the four kombucha beverages and their controls. Error bars represent the SEM

TPC assay, ORAC assay, and DPPH radical scavenging assay data of the broths are presented in Figure 3. The TPC of kombucha samples did not statistically significantly increase (*p *<* *.05) with the fermentation time compared with the unfermented samples. This was being observed for the first time, as many of the previous studies focused on lesser (i.e., 7–10 days) of fermentation. TPC of kombucha samples shows a statistically significant difference (*p *< .05) compared with the control samples, except in weeks 2, 3, 4, and 6. Tea 3—F and 4—F showed statistically significantly higher (*p *<* *.05) TPC in week 3 when compared with the initial values. Two outliers were observed as displayed in Figure 3a. Tea 3—F displayed a higher TPC value in week 3. The reduction in TPC may be due to the utilization of phenolic compounds by the tea fungus. The sudden increases in the TPCs could have coincided with the increases in the microbiological activities which was not an aspect investigated in this study. Nevertheless, the quantity of the TPC did not always determine the antioxidant activities of Kombucha, whereas the types of metabolites produced might have had the key effect instead (Chu & Chen, 2006; Greenwalt et al. 1998).

The ORAC values of kombucha samples had not statistically significantly (*p *<* *.05) increased with the fermentation time compared with the unfermented samples. The ORAC value of the kombucha beverages prepared using a higher concentration of tea dust (Tea 3—F, Tea 4—F) displayed a statistically significantly higher (*p *<* *.05) antioxidant activity compared with the other samples of lower tea concentrations. According to the mean separation, Tea 4—F had a statistically significantly higher (*p *<* *.05) ORAC value than other samples. Tea 3—F shows the highest antioxidant activity in week 5. However, the increase in the ORAC values as the fermentation progressed was gradual, when compared with previous studies such as that by Chu and Chen (2006) [study of 15 days] and Jayabalan et al. (2008) [8 days]. This may possibly be because the antioxidants had lost their antioxidant properties due to prolonged exposure to oxidation.

A statistically significant difference (*p *<* *.05) of DPPH of fermented samples was not observed on day 0, day 1, week 1, and week 2 compared with the control samples. Overall, the DPPH scavenging properties of fermented samples had statistically significantly increased (*p *<* *.05) with the fermentation time, which indicated that the antioxidant activity in terms of the DPPH radical scavenging potential was statistically significantly decreasing (*p *<* *.05). Reiss (1994) and Blanc (1996) have shown that the composition of different kombucha beverages is greatly affected by the individual tea fungal mats being used. This probably results from the variability of the normal microflora found in different tea fungus samples (Chen & Liu, 2000; J'unior et al., 2009; Jayasekera et al., 2011). Moreover, Malbaˇsa et al. (2011) reported that the application of different kombucha starters causes a development of different antioxidant activities on both substrates.

## 4 CONCLUSIONS

Overall, the kombucha samples displayed a decrease in the antioxidant activity during the 2 months of fermentation, which was suggestive that the functional properties of the beverage had decreased. Nevertheless, samples containing higher concentrations of tea displayed a higher antioxidant activity, as expected. Although it may be perceived as a preliminary study, the results obtained herein are the first of its kind given that there have been no systematic reports to date on investigating the effects of extended periods of fermentation of Kombucha. Additionally, although not investigated in this study, it is possible that prolonged fermentation may result in the accumulation of organic acids, which might reach harmful levels for direct consumption. Further studies are required to investigate this aspect. However, the study can be used as a platform to carry out more studies in the future based on different starter cultures/microbial compositions and their effect on changing the antioxidant properties of kombucha beverage during various periods of fermentation.

## ACKNOWLEDGMENTS

The authors are grateful to the financial and analytical support rendered by the National Institute of Fundamental Studies, Hantane Road, Kandy, Sri Lanka.

## DISCLOSURE OF INTERESTS

The authors declare no conflict of interests, financial, or otherwise.
